# Small quantities of respiratory syncytial virus RNA only in large droplets around infants hospitalized with acute respiratory infections

**DOI:** 10.1186/s13756-021-00968-x

**Published:** 2021-06-30

**Authors:** Jasmin S. Kutter, Dennis de Meulder, Theo M. Bestebroer, Jeroen J. A. van Kampen, Richard Molenkamp, Ron A. M. Fouchier, Jérôme O. Wishaupt, Pieter L. A. Fraaij, Sander Herfst

**Affiliations:** 1 Department of Viroscience, Erasmus University Medical Center, Dr. Molewaterplein 50, 3015 GE Rotterdam, The Netherlands; 2 Department of Pediatrics, Reinier de Graaf Hospital, Delft, The Netherlands; 3 Department of Pediatrics, Subdivision Infectious Diseases, Immunology and Rheumatology, Erasmus MC - Sophia, Rotterdam, The Netherlands

**Keywords:** Air sampling, Viable six-stage Andersen cascade impactor, Respiratory syncytial virus, Transmission routes, Droplet transmission

## Abstract

**Background:**

Respiratory syncytial virus (RSV) is a major cause of respiratory tract infections in young children. The predominant transmission routes for RSV are still a matter of debate. Specifically, it remains unclear if RSV can be transmitted through the air and what the correlation is between the amount of RSV in nasopharynx samples and in the air.

**Methods:**

The amount of RSV in the air around hospitalized RSV infected infants in single-patient rooms was quantified using a six-stage Andersen cascade impactor that collects and fractionates aerosols and droplets according to size. RSV shedding in the nasopharynx of patients was followed longitudinally by quantifying RSV RNA levels and infectious virus in nasopharyngeal aspirates. Nose and throat swabs of parents and swabs of the patient’s bedrail and a datalogger were also collected.

**Results:**

Patients remained RSV positive during the air sampling period and infectious virus was isolated up to 9 days post onset of symptoms. In three out of six patients, low levels of RSV RNA, but no infectious virus, were recovered from impactor collection plates that capture large droplets > 7 μm. For four of these patients, one or both parents were also positive for RSV. All surface swabs were RSV-negative.

**Conclusions:**

Despite the prolonged detection of infectious RSV in the nasopharynx of patients, only small amounts of RSV RNA were collected from the air around three out of six patients, which were primarily contained in large droplets which do not remain suspended in the air for long periods of time.

## Background

Respiratory syncytial virus (RSV) is a major cause of lower respiratory tract infections (LRTIs) in young children, that may be severe [1, 2]. Two antigenically different subtypes of RSV, A and B, often co-circulate, but usually one subtype predominates [3–7]. By the age of two, nearly all children have been infected with RSV at least once [8, 9]. For 2015 it was estimated that approximately 33 million children under the age of 5 suffered from an LRTI caused by RSV worldwide, of which 3.2 million required hospitalization, resulting in almost 30.000 in-hospital deaths [2].

Respiratory viruses can be transmitted via different transmission routes: via direct contact, e.g. through handshaking with an infected person, via indirect contact by touching contaminated surfaces, or via the air through droplets and/or aerosols that are expelled by an infected person [10]. Droplets quickly settle on the ground or objects in near vicinity of the source, while aerosols are small enough to remain suspended in the air for prolonged periods of time and can infect susceptible individuals further away from the source. For this reason, depending on the transmission properties of the pathogen, droplet or aerosol precautions are implemented [10].

To date, it has been widely accepted that short distances or close contact between individuals are needed for efficient RSV transmission, and as a result, contact and droplet precautions are implemented in infection prevention guidelines globally [10]. However, the scientific data to support these guidelines is scarce, and often contrasting [10]. In the 1980s it was demonstrated that healthy individuals only became infected upon self-inoculation after touching contaminated surfaces, or through close contact with infected infants, but not by solely sitting in the same room at a distance of > 1.8 m away from the patient’s bed [11]. These observations were later supported by several air sampling studies, in which RSV was detected infrequently, or not at all, in the air around infected patients [12–15]. In contrast to these studies, Aintablian and colleagues were able to collect RSV RNA from the air around RSV infected patients between 0.3 and 7 m away from the patient’s head, with a higher likelihood of RSV detection close to the patient [16]. Other researchers recently collected large quantities of infectious RSV in the air around RSV-infected children in a pediatric ward, up to 5 m from the head of an index case [17].

Because of these conflicting experimental data, the likelihood of RSV being transmitted through the air is still unknown. Here, the amount of RSV in the air around infants (< 2 years) hospitalized with RSV infections was quantified longitudinally and correlated to the RSV load in upper respiratory tract samples of these patients. With a six-stage Andersen cascade impactor that collects droplets and aerosols according to size, RSV RNA was collected from the air around three out of six infants and was found to be predominantly present in droplets > 7 μm. We did not detect infectious virus in any air fractions and did not detect RSV RNA in finer aerosols, whereas such finer aerosol fractions did contain rhinovirus RNA.

## Materials and methods

### Patients

The study was conducted during three consecutive winter seasons (November 2017–April 2020) at the department of pediatrics at the Reinier de Graaf Hospital, Delft, The Netherlands. Hospitalized children aged between 0 and 2 years with laboratory-confirmed RSV-A or RSV-B infections were both eligible. However, during these three seasons, only RSV-B positive patients were detected in the study. Patients were excluded if they were older than 2 years, or if signed informed consent was not obtained. For practical reasons, immunocompromised patients and patients that were experiencing symptoms for more than a week prior to hospitalization were not included. Patients with other co-morbidities, with viral co-infections, or use of medication, including antivirals, were not excluded, but this information was documented together with other clinical data obtained from the patient’s medical record using a standardized case record form, specifically developed for this study. Patients were hospitalized in single-patient rooms with an additional bed for parents. All patient rooms had a ventilation rate of 2 air changes per hour (ACH) with a fresh air transportation rate of 100 m^3^/h. Temperature and relative humidity were recorded every five minutes during the whole air sampling period using EL-GFX-2 dataloggers (Lascar Electronics Inc.).

### Sample collection

For the collection of RSV from the air, the six-stage Andersen cascade impactor (Thermo Scientific™) was used [18]. The cascade impactor operates at a flow rate of 28.3 L per minute (LPM) and collects droplets and aerosols according to size in six different stages: Stage 1 (> 7.0 μm), stage 2 (7.0–4.7 μm), stage 3 (4.7–3.3 μm), stage 4 (3.3–2.1 μm), stage 5 (2.1–1.1 μm) and stage 6 (1.1–0.65 μm). The air inlet of the cascade impactor was positioned approximately 1 m away from the patient’s head at a height of 109 cm. Air was sampled daily for 30 min, starting the day after the informed consent was obtained, until discharge or a maximum of five days. To prevent contamination of samples, air sampling was performed in the morning, prior to routine care that may involve aerosol generating procedures like nasopharyngeal aspiration. Droplets and aerosols were impacted onto collection plates filled with an in-house developed semi-solid gelatin layer, as previously described [19]. The gelatin layer was prepared from commercial gelatin sheets (10 mg/ml; Dr. Oetker) dissolved in virus transport medium (VTM). VTM consisted of Minimum Essential Medium (MEM) – Eagle with Hank’s BSS and 25 mM Hepes (Lonza), glycerol 99 % (Sigma Aldrich), lactalbumin hydrolysate (Sigma Aldrich), 10 MU polymyxin B sulphate (Sigma Aldrich), 5 MU nystatin (Sigma Aldrich), 50 mg/ml gentamicin (Gibco) and 100 IU/ml penicillin 100 µg/ml streptomycin mixture (Lonza). To avoid high dilution factors, each collection plate was first filled with 32 ml of 2 % agarose (Roche) as a bottom layer on which 9 ml of the semi-solid gelatin was pipetted. Subsequently, plates were stored at + 4 °C for a maximum of 4 days.

On the last day of air sampling, surface swabs were taken using Copan flocked swabs (Copan Diagnostics Inc.) from the datalogger and the bed rail on the side where the cascade impactor was located. From the first day of air sampling onwards, nasopharyngeal aspirates (for decongestion) were obtained from patients during routine clinical care if available, and otherwise aspirates or nose swabs were taken specifically for this study if consent was given by the parents. From each parent, nose or throat swabs (either Copan eSwab® or Copan flocked swabs (Copan Diagnostics Inc.)) were taken on the first and last day of air sampling, when possible, to estimate their contribution to viral shedding in the air.

### Sample processing

The collection plates with gelatin from the cascade impactor were processed after sampling by adding 6 ml of prewarmed VTM, followed by a 30 min incubation at 37 °C to dissolve the gelatin layer and harvesting of the samples [19]. Nose and throat swabs of parents were collected in Amies (eSwab®; Copan Diagnostics Inc.) or universal transport medium (UTM; flocked swabs; Copan Diagnostics Inc.). VTM was added to the nasopharyngeal aspirates of patients and nose and throat samples of parents to reach a total volume of 6 ml, after which nasopharyngeal aspirates were centrifuged at 500 G for 5 min to remove cell debris. All samples were subsequently aliquoted and stored at + 4 °C for a maximum of 4 days until further analysis. An additional vial of each sample was stored at -80 °C. The surface swabs were collected in UTM (flocked swabs; Copan Diagnostics Inc.), and stored in 25 % sucrose at -80 °C. At the end of the study, the samples were thawed and RNA was extracted followed by qRT-PCR analysis.

### Cells

Hep-2 cells were cultured in Dulbecco’s Modified Eagle Medium (DMEM, Lonza or Gibco) supplemented with 10 % fetal bovine serum (FBS) (Greiner or Atlanta Biologicals), 100 IU/ml penicillin 100 µg/ml streptomycin mixture (Lonza), 200 mM L-glutamine (Lonza), 1.5 mg/ml sodium bicarbonate (Lonza), 10 mM HEPES (Lonza) and 0.25 mg/ml fungizone (Invitrogen). The cells were maintained at 37 °C and 5 % CO_2_.

### RNA extraction and qRT-PCR

RNA was extracted from the samples using the MagNA Pure LC Total Nucleic Acid Isolation Kit (Roche) and subjected to qRT-PCR analysis for the detection of RSV-B RNA, and in case of the rhinovirus (RV) co-infected patients also for RV RNA [20]. For this purpose, 20 µl of extracted virus RNA was amplified in a final volume of 30 µl, containing 7,5 µl 4xTaqMan Fast Virus 1-Step Master Mix (Life Technologies) and 1 µl Primer/Probe mixture [20]. Amplification was performed using the following protocol: 5 min 50 °C, 20 s. 95 °C, 45 cycles of 3 s. 95 °C and 31 s. 60 °C. For all samples, a cycle threshold (Ct) value of > 40 was considered negative.

### Virus titration

RSV-B-positive samples were titrated on Hep-2 cells for the quantification of infectious virus, as defined by the culturability of RSV. Briefly, Hep-2 cells were grown to confluency in 96 well plates overnight. Subsequently, cells were spin-inoculated (15 min, 2000 rpm) with 100 µl of 10-fold serial dilutions of RSV-B positive samples and incubated at 37 °C, 5 % CO_2_. One hour after inoculation, cells were washed once and cultured in serum-reduced (2 %) Dulbecco’s Modified Eagle Medium (DMEM, Lonza) supplemented with 100 IU/ml penicillin 100 µg/ml streptomycin mixture (Lonza), 200 mM L-glutamine (Lonza), 1.5 mg/ml sodium bicarbonate (Lonza), 10 mM HEPES (Lonza) and 0.25 mg/ml fungizone (Invitrogen). After 7 days of incubation, positive samples were identified by immunofluorescence assays using a FITC labelled polyclonal antibody directed against RSV (Fisher Scientific). Infectious virus titers were calculated from four replicates as tissue culture infective dose (TCID_50_) by the Spearman-Karber method.

## Results

### Clinical setting and patient demographics

Six infants with ages ranging from 10 days to 7 months (median age 2 months) were included over the course of three consecutive winter seasons (Nov 2017–Mar 2020), of whom two were female and four were male (Table 1). No comorbidities were reported, however, patient 1 was born at a gestational age of 36 weeks. All patients had RSV-B confirmed infections with the majority of infections manifesting as bronchiolitis and/or dyspnea. Two patients were diagnosed with a rhinovirus (RV) co-infection at the day of hospital admission. All infants received non-invasive positive airway pressure ventilation with supplemental oxygen. Standard treatment was only supportive aimed at relieving symptoms (Table 1). All infants were hospitalized in single-patient rooms together with one in-rooming parent. Ambient room temperature and relative humidity were monitored in each room and were on average 21.7 °C and 45.5 %, respectively (Table 2).

**Table 1:** Patient demographics and medical information

Patient	Sex	Age	Weight (kg)	Co- infection	Symptom	Started with vaccination program*	Suppl. oxygen	Medication^$^	Antibiotics	Other^§^
**1**	M	2 months^¥^	4.7	–	Dyspnea	No	Yes	Ipratropium (0.25 mg) Salbutamol (2.5 mg) Xylometazoline (0.25 mg/ml) Paracetamol (60 mg)	No	No
**2**	F	1 month	5.0	–	Bronchiolitis	No	Yes	Xylometazoline (0.25 mg/ml) Paracetamol (60 mg)	No	Vitamin K oil droplets
**3**	M	10 days	3.8	–	Sinusitis	No	Yes	Xylometazoline (0.25 mg/ml) Paracetamol (60 mg)	Amoxicillin (500 mg/ml) Cefotaxime (500 mg/ml)	No
**4**	M	2 months	6.4	RV	Dyspnea	No	Yes	Xylometazoline (0.25 mg/ml) Paracetamol (120 mg)	No	Vitamin K oil droplets Cholecalciferol (10 µg)
**5**	M	7 months	11.1	RV	Bronchiolitis & Tachypnea	Yes	Yes	Xylometazoline (0.25 mg/ml) Paracetamol (240 mg) Hypertonic saline inhalation (2.5 mg/ml)	No	Cholecalciferol (10 µg)
**6**	F	3 months	6.0	–	Bronchiolitis & Dyspnea	Yes	Yes	Ibuprofen (20 mg/ml) Paracetamol (120 mg) Hypertonic saline inhalation (0.9 mg/ml)	No	Cholecalciferol (10 µg)

Standard medical treatment was only supportive and aimed at relieving symptoms. (¥) born premature, (*) according to Dutch vaccination program, ($) was given if needed, (§) was given once a day

**Table 2:** Overview of environmental conditions and Ct values in air samples and samples from patients and parents

Patient	Days between symptom onset and 1st day of sampling	Ambient room temperature (°C)		Room relative humidity (%)		Ct value of RSV air sample (day)	Ct value of RV air sample (day)	Ct value of father for RSV (day)	Ct value of mother for RSV (day)
		Average	SD	Average	SD				
1	Unknown	21.8	0.1	47.9	3.9	34.9 (2)	–	28.2 (4) 31.4 (5)	21.4 (1) 33.6 (5)
2	4	22.0	0.2	48.8	3.9	33.5 (2) 35.8 (5)	–	Neg (1) Neg (4)	Neg (1) 35.5 (4)
3	6	21.7	0.1	42.2	2.1	34.9 (2)	–	Neg (1)	Neg (1) Neg (5)
4	6	21.8	0.4	43.5	1.9	Neg	35.7 (2) 35.3 (2) 37.8 (4) 35.6 (5)	31.8 (1) 33.3 (5)	Neg (1) Neg (5)
5	5	21.5	1.0	45.6	3.4	Neg	Neg	22.4 (1) 20.4 (1)	Neg (1) Neg (3)
6	7	21.7	0.1	44.6	3.9	Neg	–	Neg (3)	Neg (2)

### RSV load in patients and parents

**Fig. 1:**
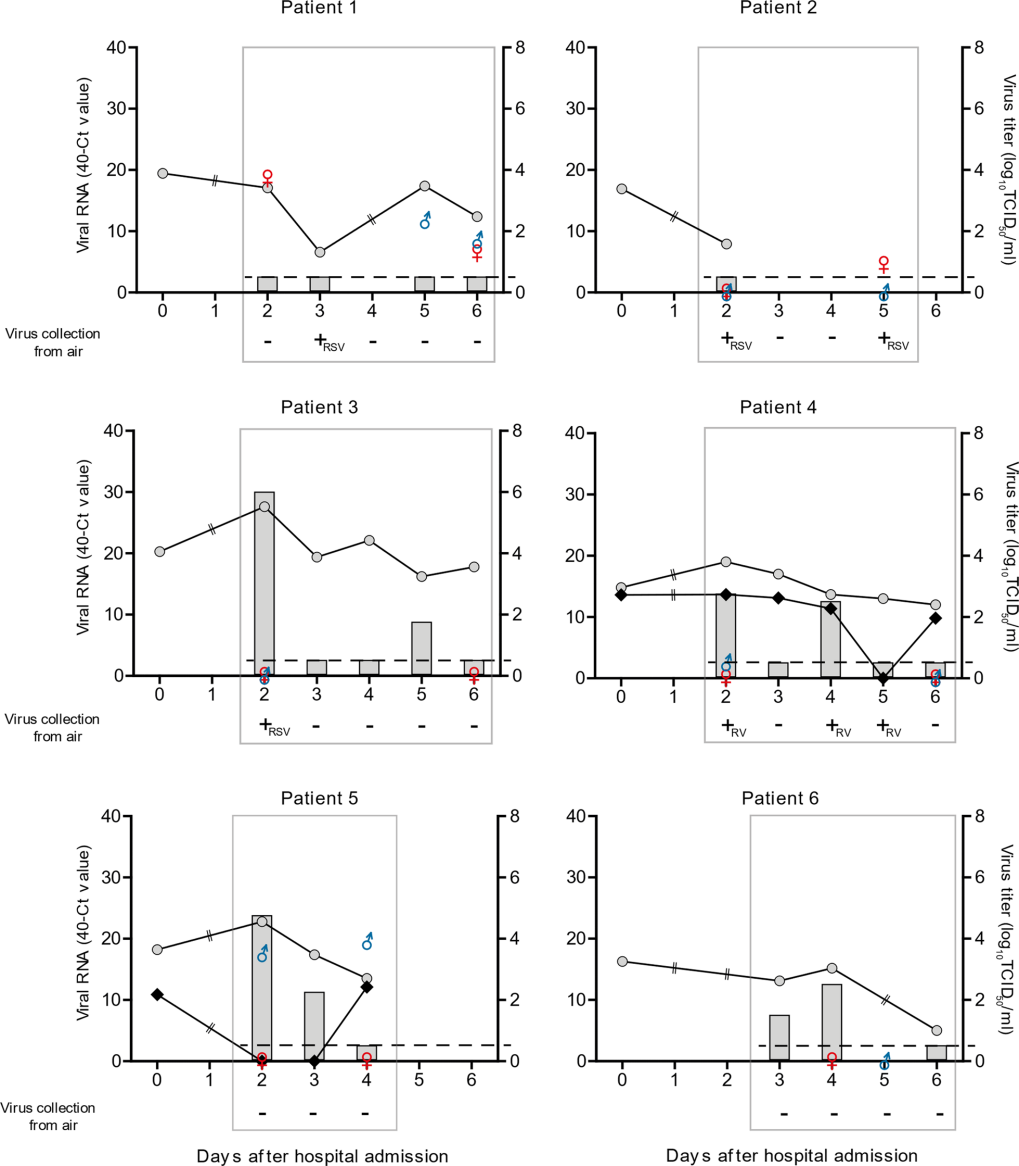
Graphs representing the viral load in 6 infants, their parents, and air samples. Grey circles represent RSV-B RNA, black diamonds RV RNA (left Y axis) and grey bars RSV-B titers (right Y axis) of nasopharyngeal aspirates of patients. Circles and diamonds are replaced by a double line at days on which no sample from the patient was obtained. Gender symbols represent total RSV-B RNA of mother (red venus) and father (blue mars). Dashed horizontal lines indicate the detection limit of virus titrations. Grey rectangles mark the period during which air sampling was performed. Plus and minus signs indicate if air samples were positive or negative on the day of air sampling

To study the virus shedding kinetics in patients, both the RSV RNA levels, as well as the amount of infectious virus, were determined in the nasopharyngeal aspirates that were collected over time. For patient 2, only one nasopharyngeal aspirate was obtained, because the parents did not consent to obtain an aspirate solely for the purpose of this study. For this reason, it is unknown if this patient remained RSV positive during the sampling period. However, the other five patients were qRT-PCR positive during the entire sampling period (Fig. 1). In four patients, infectious RSV was isolated from the nasopharyngeal aspirates on more than one day. For RV co-infected patients, aspirates were RV-positive by qRT-PCR on all days except on day 5 (Patient 4) and day 2 and 3 (Patient 5) after hospital admission (Fig. 1).

Since parents stayed with their children during hospitalization, it was possible that they became infected as well. For this reason, nose or throat swabs were collected from the parents. For four patients, at least one parent tested positive for RSV-B by qRT-PCR (Fig. 1). For patient 1, both parents were found to be RSV-B positive by qRT-PCR. All parents of RV co-infected patients tested negative for RV. High levels of RSV RNA, similar to those in the patients, were detected in swabs collected from the mother of patient 1 and the father of patient 5, but no infectious virus was detected (Table 2).

### RSV in air and on surfaces

Air sampling in patient rooms was started 4 to 7 days (median 6 days, Table 2) after the onset of symptoms. In three out of six patient rooms, RSV RNA was collected from the air on at least one day (Fig. 1). For patient 1 and 3, RSV RNA was detected on day 3 and 2 after hospital admission, respectively. In the room of patient 2, RSV RNA was detected in air samples intermittently on days 2 and 5 after admission. All RSV RNA positive air samples originated from stage 1 with the largest droplet particle size (> 7 μm). The quantities of virus RNA retrieved from the RSV-positive samples were low (Ct values ranging between 33.5 and 35.8) and no infectious virus was detected (Table 2).

For one of the two patients co-infected with RV, air samples were positive for RV RNA (patient 4). Stage 1 (particles > 7 μm) was positive for RV RNA on day 2 and stage 3 (particles between 4.1 and 3.3 μm) was positive on days 2, 4, and 5 after admission. RV RNA levels were also low, with Ct values ranging between 35.3 and 37.8 (Table 2; Fig. 1).

To investigate if RSV and RV were also present on surfaces, the bedrail on the side of the cascade impactor and the datalogger which was placed approximately 1.5–2 m from the patient’s head were swabbed on the day of discharge (Table 3). A bedrail swab of patient 6 is missing because the bedrail had already been disinfected and cleaned before a sample could be obtained. No RSV or RV RNA was detected in any of the surface swabs by qRT-PCR.

**Table 3:** Detection rate of RSV and RV in all collected samples

Sample	# of samples obtained	# of samples positive	
		Total viral RNA	Infectious virus
Nasopharyngeal aspirates	21	21	8
Nose swabs parents	21	8	–
Mother	11	3	–
Father	10	5	–
Air samples	34	7	–
RSV	26	4	–
RV	8	3	–
Surface swabs	11	–	–
Bedrail	5	–	–
Datalogger	6	–	–

## Discussion

Despite the substantial impact of RSV globally, it is still unclear via which routes RSV is primarily transmitted and if and how long infectious virus is shed by infected individuals. Here, we determined the amount of RSV in the air around hospitalized infants, in correlation with the viral load in their upper respiratory tract over time. We demonstrated that despite the presence of infectious RSV in nasopharyngeal samples of infants, only low amounts of RSV RNA, but no infectious virus, were detected in the air around three out of six patients. RSV RNA was only detected in large (> 7 μm) droplets.

For two of these patients, one or both parents also tested positive for RSV, so they may have contributed to the RSV RNA quantities collected from the air. For the third patient in whose room RSV was collected from the air, both parents were RSV negative, and therefore the RSV RNA must have been expelled by the patient.

For one patient who was co-infected with RV, low amounts of RV RNA were collected from the air on multiple days. Except for one positive sample that was recovered from stage 1 (droplets > 7 μm), the remaining three RV positive air samples were consistently recovered from stage 3 of the cascade impactor. This stage collects aerosols in the size range of 3.3 to 4.7 μm, indicating that at the time of air sampling RV RNA was contained in smaller particles than RSV RNA. Remarkably, on a day that the airway samples collected from the patient and both parents were negative for RV RNA, an air sample turned out to be positive. It is unclear if virus was still shed by the patient or the parents, but from an anatomical site of the respiratory tract that was not sampled, or that the air was contaminated by hospital personnel that was present that day.

Air sampling was only started a few days to one week after symptom onset during the late phase of infection, which may explain the low quantities of RSV and RV RNA collected from the air (Table 2). In several other studies, in which various air samplers were used, also low numbers of RSV RNA positive air samples were reported, with a detection rate ranging from 2.3 to 31.8 % [12–14, 16]. Moreover, in a recent study by Chamseddine et al., none of the collected air samples around RSV infected patients were positive for RSV RNA, while half of the air samples collected around influenza A virus-infected patients were positive for influenza virus RNA. However, attempts to isolate infectious influenza virus from these samples were not successful [15].

Contrasting results were previously reported by Kulkarni et al., where air sampling around infants with RSV-confirmed bronchiolitis in general wards of a pediatric hospital resulted in the collection of high amounts of infectious RSV from the air [17]. Although viral quantities in the air decreased with increasing distance to the patient’s head, up to 10^5.6^ plaque forming units (PFU) of infectious RSV were still collected 5 m away from the patient’s head. As in the present study, Kulkarni and colleagues used a six-stage Andersen cascade impactor, however, liquid medium was used as a collection medium. We have recently shown in an in-vitro set-up that the collection of infectious virus using liquid medium is less efficient than when semi-solid gelatin is used (as in the current study), so this does not explain the differences in collected amounts of RSV between the studies [19]. It should be noted that the Andersen cascade impactor was designed and validated with solid impaction media rather than liquids [18].

The small amounts of RSV RNA detected in large droplets and the total absence of infectious RSV in the air around infected infants as presented here, and the low detection rates of RSV RNA in the air in most other studies using various air samplers, indicate that transmission via the air is unlikely to be a route by which RSV spreads in the population. This observation is also supported by the fact that room-sharing of RSV infected and non-infected patients did not seem to influence the risk of nosocomial infections [21]. In addition, wearing gowns and gloves, and adhering to strict hygiene has been shown to reduce the risk of nosocomial RSV transmission considerably, further indicating that aerosol transmission is not efficient and possibly negligible in this context [22, 23].

To investigate the possibility of RSV transmission through fomites, we also took surface swabs of the bedrails and dataloggers on the last day of air sampling. In none of the surface swabs, RSV RNA was detected by qRT-PCR, which is in contrast to the study of Wan et al., where RSV RNA was detected on various objects [12]. A reason for the conflicting results might be the timepoint when the surface swabs were taken. Wan and colleagues took surface swabs shortly after the admission of patients. In our study, surface swabs were only taken on the last day of air sampling, during the late stage of RSV infection when RSV RNA levels in the patients had already decreased.

## Conclusions

In conclusion, we here demonstrate that despite the shedding of infectious RSV from the nasopharynx of hospitalized infants, no or only low amounts of RSV RNA were detected, but no infectious RSV was detected in any of the collected air samples. RSV RNA was only detected in large droplet samples on only a limited number of days. These results suggests that in the current hospital setting, RSV transmission through the air at later stages of infection was negligible and that the implementation of contact and droplet precautions, as currently employed in most hospitals, is sufficient.

## Data Availability

All data and materials are available from the corresponding author (S.H.) on reasonable request.

## Abbreviations

ACH: Air changes per hour
Ct: Cycle threshold
DMEM: Dulbecco’s Modified Eagle Medium
LPM: Liters per minute
LRTI: Lower respiratory tract infection
MEM: Minimum Essential Medium
PFU: Plaque forming unit
qRT-PCR: real time quantitative reverse transcription polymerase chain reaction
RSV: Respiratory Syncytial Virus
RV: Rhinovirus
TCID_50_: 50% Tissue culture infectious dose
UTM: Universal transport medium
VTM: Virus transport medium
